# Relationships between upper extremity neuromuscular function and patient-reported outcomes among individuals with a history of glenohumeral labral repair

**DOI:** 10.1371/journal.pone.0338260

**Published:** 2025-12-12

**Authors:** Katsumi Takeno, Christopher D. Ingersoll, Neal R. Glaviano, Sadik Khuder, Grant E. Norte

**Affiliations:** 1 Department of Kinesiology, University of North Georgia, Dahlonega, Georgia, United States of America; 2 School of Medicine, University of North Carolina Chapel Hill, Chapel Hill, North Carolina, United States of America; 3 Department of Kinesiology, University of Connecticut, Storrs, Connecticut, United States of America; 4 School of Medicine, University of Toledo, Toledo, Ohio, United States of America; 5 College of Health Professions and Sciences, University of Central Florida, Orlando, Florida, United States of America; Adnan Menderes Universitesi, TÜRKIYE

## Abstract

**Background:**

Patient-reported outcomes (PROs) are essential components of contemporary healthcare practices, which allow clinicians to effectively monitor clinical outcomes. Objectively measured neuromuscular impairments have been reported among individuals with glenohumeral labral repair, yet their relationship with PROs is unclear. Understanding those relationships would help clinicians explain clinical meaning of such impairments from a patient-oriented perspective. The objective of this study was to determine the relationships between objective metrics of upper extremity neuromuscular function and PROs in individuals with glenohumeral labral repair.

**Methods:**

16 individuals with a history of glenohumeral labral repair (13 males/3 females, age: 24.1 ± 5.5 years, time from surgery: 36.3 ± 33.3 months) volunteered. Neuromuscular functions, characterized by shoulder abduction and wrist flexion maximal voluntary isometric contraction (MVIC) torque, Hoffmann reflex (spinal pathway), and active motor threshold (AMT, corticospinal pathway) of the upper extremity muscles, were assessed bilaterally. Pain, physical activity level, Disability of Arm, Shoulder and Hand (DASH) questionnaire, Oxford Shoulder Score (OSS), and Veteran’s Rand 12-Item Health Survey (VR-12) were used to quantify self-reported upper extremity function and global health, encompassing both mental and physical components. Bivariate correlations and multiple linear regression were used to identify the variance explained by neuromuscular outcomes in each PRO.

**Results:**

Involved limb wrist flexion MVIC torque moderately correlated with DASH (*r* = −.523, *p* = .045). AMT of middle deltoid moderately correlated with OSS (ρ = .570, *p* = .021). Age and current activity level explained 73.2% variance in the VR-12 physical component (adjusted *R*^*2*^ = .732, *p* < .001). Age moderately correlated with the VR-12 mental component (ρ = .550, *p* = .027).

**Conclusions:**

Greater involved limb wrist flexor strength, corticospinal excitability of the middle deltoid, as well as greater age and lesser activity level explained better perceived function and health. These results support the clinical relevance of muscle strength and neural function to patient recovery from glenohumeral labral repair.

## Introduction

Glenohumeral labral tears are one of the most common pathological conditions affecting the upper extremities among physically active populations [1–5]. Persistent shoulder pain and instability often require surgical repair to resume demanding overhead activities [6]. Failure to address common symptoms can adversely affect activities of daily living, athletic performance and overall quality of life. However, the assessment of clinical outcomes among individuals with glenohumeral labral repair is complex and multifaceted. Common assessment practices are based on the resolution of impairments, return to preinjury levels of physical performance, patient perceptions of readiness to return to sport, and satisfaction with functional outcomes [7]. Despite such practices, individuals with glenohumeral labral repair exhibit consistent deficits in shoulder strength years after surgery [8], suggesting a need to optimize clinical outcomes.

While impairments in muscle function are common in this population, and resolution of those impairments remains a primary focus of rehabilitation, patient-reported outcomes (PRO) have become essential components of contemporary patient-centered healthcare. Subjective measures of function and health allow clinicians to assess the effectiveness of rehabilitation and track clinical outcomes, which serve as the foundation of evidence-based health care. For this purpose, PROs play a critical role in clinical and research aspects of orthopedic surgery and rehabilitation as a measure of quality, particularly those that relate to commonly observed physical impairments, and instruments that can differentiate subtle changes are essential.

For individuals with shoulder injury or surgery, several PROs are commonly used to assess regional function. The Disabilities of the Arm, Shoulder and Hand (DASH) questionnaire is a self-administered region-specific outcome instrument developed as a measure of self-reported upper extremity disability and symptoms [9]. The validity and reliability of the DASH have been well established to assess effectiveness of interventions among adult patients who have undergone upper extremity orthopedic surgery [10]. The Oxford Shoulder Scores (OSS) is another self-reported instrument used to quantify perceived change of functional status in the operated shoulder [11]. Since the DASH considers both limbs, it may be vulnerable to compensatory movement strategies, potentially overestimating function. For example, using a phone could be performed by an uninvolved limb with no difficulty but the score might not accurately reflect the individual’s perceived function of the involved shoulder. For this reason, pairing the DASH with the OSS can provide more robust information to researchers and clinicians about general upper extremity and shoulder-specific functioning of the recovering limb.

While most PROs are disease/injury specific and focus on regional function of shoulder and upper extremity, generic PROs aim to assess global health status by measuring multiple domains of quality of life and well-being, which are valuable in comparing health across a range of disease/injury processes [12]. Since health encompasses not only the absence of disease and infirmity but also the presence of physical, mental, and social well-being, quality of life issues have become steadily more important in health care practice and research [13]. The Veteran’s Rand 12-Item Health Survey (VR-12) is one of global health related quality of life measures that is quantified by physical component scores (PCS) and mental component scores (MCS), inclusive of general health, emotions, physical activity, pain, and personal feelings following injury [14].

From a clinical perspective, physical impairments following musculoskeletal injury or surgery are routinely managed by addressing modifiable factors, and thus muscle function represents a primary clinical priority during rehabilitation following glenohumeral labral repair. Although clinicians attempt to target functional impairments with traditional rehabilitation, such as progressive strengthening exercises, persistent muscle weakness [8] or altered patterns of muscle activation [15–17] are reported in this population, highlighting the need to explore lesser known origins of muscular impairments [18]. The glenohumeral joint requires coordinated activation of the shoulder and periscapular musculature to achieve functional stability. During controlled shoulder movements, scapular muscles (e.g., upper trapezius) provide sustained postural support and stabilization of the glenohumeral joint, while arm muscles (e.g., middle deltoid) execute various osteokinematic motions [19]. In addition, muscular impairments exist distal to the site of injury (e.g., flexor carpi radialis) as well in this population, suggesting a need to explore beyond the shoulder complex [20].

A growing body of evidence has shown that neuromuscular alterations following traumatic joint injury or orthopedic surgery are thought to be associated with impairment of muscle functions. Recent work has extended our understanding of post-traumatic muscular impairments in those with glenohumeral labral repair by demonstrating persistent muscle weakness and suppressed neural excitability among muscles crossing the injured glenohumeral joint as well as those proximal and distal to the shoulder [20]. These findings support previously observed neuromuscular abnormalities in patients with rotator cuff injury [15–17] and non-traumatic shoulder instability [21]. Collectively, neuromuscular impairments observed in individuals with upper extremity pathologies may explain persistent muscle weakness or dysfunction reported following injury or surgery. However, their associations with patient-reported function and well-being in individuals with glenohumeral labral repair remain unclear.

Understanding if objective measures of neuromuscular function matter from a patient-oriented perspective is important for researchers and clinicians to develop evidence-based assessment and intervention protocols that allows them to set priority in injury management and better inform clinical decision making. Therefore, the purpose of this study was to determine the relationships between objective measures of neuromuscular function in the shoulder and upper extremity muscles, and patient-reported outcomes in individuals with a history of glenohumeral labral repair. We hypothesized that greater muscle strength, spinal- and supraspinal-level neural excitability would be associated with better patient-reported shoulder and upper extremity function, as well as global health.

## Materials and methods

We used a cross-sectional design to assess the relationships between objective measures of shoulder and upper extremity neuromuscular function and patient-reported function in individuals with a history of glenohumeral labral repair. Explanatory variables included shoulder abduction and wrist flexion maximal voluntary isometric contraction (MVIC) torque. Active motor thresholds (AMT) were measured for the upper trapezius, middle deltoid, and flexor carpi radialis muscles. Hoffmann reflexes (H-reflex) were only measured in the flexor carpi radialis due to the difficulty in accessing peripheral nerves that innervate the upper trapezius and middle deltoid. Dependent variables included the DASH (upper extremity disability and symptoms), OSS (shoulder-specific symptoms), and VR-12 (global health: physical and mental components) scores.

### Participants

16 individuals with a history of primary, unilateral glenohumeral labral repair between the ages of 19–36 years old participated in this study. Participants were recruited in the local university community from November 2018 to March 2020. To be eligible, individuals must have undergone a primary, unilateral labral repair at minimum of 6 months before participation without postoperative complications. The minimum post-surgical time was chosen as it was expected to be sufficient to show improvement after surgery in most cases or actual medical clearance without limitation. Individuals were excluded if they had previous upper extremity orthopedic surgery at any point in time, upper extremity injury other than glenohumeral labral repair within 6 months, concussion within 6 months, or use of central nervous system stimulants or depressants (e.g., antispastics, anxiolytics, hypnotics, anti-epileptics). All participants were screened for the use of transcranial magnetic stimulation (TMS) according to the safety and ethical guidelines outlined by the International Federation of Clinical Neurophysiology and the National Institutes of Neurological Disorders and Stroke [22]. The Institutional Review Board for Biomedical Research at the University of Toledo (Toledo, OH, USA) approved this study, and all participants provided written informed consent prior to enrollment.

### Procedures

Participants were asked to avoid caffeine use and strenuous exercise within 12 hours prior to testing to avoid confounding study outcomes [23]. Testing was conducted during two sessions separated by 24–48 hours. A single researcher performed all testing procedures across the duration of the study. Outcome measures were recorded bilaterally, and the order of testing was randomized based on limb dominance and muscle by the investigator rolling a die (odd number: right, even number: left, 1 or 2: upper trapezius, 3 or 4: middle deltoid, and 5 or 6: flexor carpi radialis) before session 1. Limb dominance was recorded for each participant and was determined by which limb would be used to throw a ball with.

### Participant Set-up

The area of greatest bulk of the muscle was shaved, cleaned and debrided. Two 10 mm pre-gelled Ag-AgCl surface electromyographic (EMG) electrodes (EL503, BIOPAC Systems, Inc.) were positioned over each muscle tested as recommended in the SENIAM guidelines [24] with 2.0 cm inter-electrode distance. The pairs of recording electrodes were positioned over the upper trapezius in the midpoint between the acromion and the C7 spinous process, over the greatest bulge of the middle deltoid in the direction of the line between the acromion and the lateral epicondyle of the elbow, and over the flexor carpi radialis in the anterior forearm approximately one-third the distance from the medial epicondyle to the radial styloid [25]. The area of skin where the recording electrodes were positioned was marked with a surgical marker at the end in session 1, so that we could use consistent recording electrode position in session 2.

### Session 1

#### Patient-Reported Outcomes.

Participants completed PROs prior to testing. The DASH questionnaire is a self-administered region-specific outcome instrument developed as a measure of self-rated upper extremity disability and symptoms [9]. Assessment consists of the 30 items that are scored on 5 levels of Likert scale (1 = no difficulty/symptom, 5 = extreme difficulty/symptoms). We calculated the arithmetic mean of the 30 items transformed to the scale from 0 (= no symptom/full function) to 100 (= worst symptoms/no function) for comparison [26]. The validity and reliability of the DASH have been well established (ICC: 0.77–0.98, MCID: 10 for shoulder complaints) to assess effectiveness of interventions among patients who underwent surgery (sensitivity: 0.82, specificity: 0.74) [10,27]. We also chose to use the OSS (sensitivity: 0.96, specificity: 0.83) [28] for self-rated change of functional status in the operated arm [11] in order to ensure that it did not capture global health changes, such as compensatory movement strategies, which may not be related to the affected shoulder. The sum of the 12 items on 5 levels of Likert scale provides total score from 12 (= best) to 60 (= worst). Internal reliability and test-retest reliability of the OSS have also been demonstrated previously (Cronbach’s α: 0.94, Pearson’s correlation: 0.98, no published ICC data) [29]. The VR-12 is one of global health related quality of life measures that quantified by physical component summary (PCS) and mental component summery (MCS), such as general health, emotions, physical activity, pain, and personal feelings following injury [14]. Computation of PCS and MCS is standardized to the scores in a contemporary U.S. population based on the Medical Expenditure Panel Survey, so that the score of 50 can be considered on the 50th percentile in the population, and higher PCS and MCS scores indicate better health [14]. Previous studies reported good reliability for both physical (ICC: 0.80) and mental (ICC: 0.74) health scores [30], and minimally important differences in patients following treatment of rotator cuff tears were 4.94 and 5.99, respectively [31]. We also collected participants’ current and pre-injury activity levels using a Tegner Activity Scale, and current pain was assessed using visual analog scale (VAS) at the end of the first session.

#### Muscle strength.

Participants were seated in a height-adjustable chair with the hips flexed to 85° with a belt worn at the pelvis throughout the testing. Participants completed a standardized acclimatization protocol with a series of sub-maximal trials (25%, 50%, 75% perceived effort) performed prior to recording three maximal effort trials [32]. During trials, the contralateral limb was relaxed, and the hand was placed on the lap. Maximal effort trials were repeated 3 times for each position with at least 1 minute rest between the trials. The investigator provided verbal encouragement and visually inspected any accessory movement for the use of obvious compensatory strategy for the duration of each trial. If the use of an improper technique was observed [30], the trial was excluded from analysis.

We recorded MVIC force (N) and external moment arm (m) to calculate the average MVIC torque (Nm) from 3 test trials, which was further normalized by body mass (Nm/kg) to quantify standardized muscle strength. The recording procedures for each selected muscle were as follows:

For isometric shoulder abduction strength, participants were asked to raise the arm to 90° unilaterally in the scapular plane with the elbow extended and forearms pronated, and then to maintain the position against downward manual resistance on the elbow through a hand-held dynamometer (microFET2 MMT Wireless, Hoggan Health Industries, Inc., West Jordan, UT) for 5 seconds. According to previously published studies, this technique has yielded high reliability (ICC2,1: 0.85–0.99) [33,34]. While remaining in the seated position described above, participants were positioned at 30° shoulder flexion, 45° elbow flexion, and the posterior forearm was rested on a padded armrest with supination. The wrist was maintained in neutral position where the forearm and hand were aligned in the same plane. For isometric wrist flexion strength, participants were asked to maintain a neutral wrist position against manual resistance for five seconds. Manual resistance was applied toward the direction of wrist extension on the palmar metacarpal heads through a hand-held dynamometer.

#### EMG Recording.

We recorded muscle activation simultaneously during strength testing using surface EMG to calculate the external loading needed to perform standardized pre-activation of the upper trapezius, middle deltoid and flexor carpi radialis muscles across the participants during H-reflex and TMS testing procedures in session 2. We aimed to have participants achieve sub-maximal muscle activation at 15% (upper trapezius), 20% (middle deltoid) [17] and 5% (flexor carpi radialis) [25] MVIC to standardize the level of muscle pre-activation across participants. We calculated the mean EMG amplitude from the middle three seconds of each isometric muscle contraction to identify the target EMG activity for each muscle. Following the MVIC trials, the tasks described above were repeated incrementally by adding external weight (e.g., limb weight, 1 lb, 2 lbs) until the target EMG amplitude was recorded for each muscle. EMG signals were sampled at 2000 Hz, band-pass filtered from 10–500 Hz, then rectified and smoothed using a 30-millisecond root-mean-squared sliding window in AcqKnowledge 5.0.3 software (BIOPAC Systems, Inc.). The same EMG sampling and filtering frequencies were used during H-reflex and TMS testing.

### Session 2

#### Motoneuron pool excitability.

The H-reflex was used to assess spinal-reflexive excitability of the flexor carpi radialis. Participants were asked to abstain from caffeine consumption and strenuous exercise for a minimum of 12 hours prior to session 2. Testing was completed in a quiet, dimly lit room to minimize external sensory input that may affect neural excitability at spinal level. Participants were in an upright seated position in a height adjustable chair with the shoulder flexed at 30°, the elbow flexed at 45°, and the tested forearm supported posteriorly on a padded armrest in the supinated position. The contralateral hand was placed on the lap.

A previous study [35] suggested difficulties to elicit H-reflexes from upper extremity muscles without facilitation using low-level muscle contraction. Therefore, a standardized submaximal isometric muscle contraction was performed using a dumbbell that elicited EMG amplitudes at approximately 5% MVIC (observed activation: 5.6 ± 1.8%) to facilitate the H-reflex [25,36]. Participants were instructed to maintain an isometric flexor carpi radialis contraction by loosely holding a dumbbell in the neutral wrist position as electric nerve stimulation was delivered, and then to relax the muscle immediately following each stimulus. This technique has yielded moderate test-retest reliability (ICC_3,1_: 0.66, MDC_95_: 0.11) [25].

We generated H-reflex and muscle response (M-wave) recruitment curves using techniques as previously described [37]. A stimulating bar electrode (ELSTM1, BIOPAC Systems Inc.) was placed over the median nerve in the antecubital fossa between the medial condyle of the humerus and distal biceps brachii tendon [25]. Each response was elicited using a series of 1-milisecond square wave electrical stimuli ranging 10–200 volts delivered to the median nerve via a stimulator module (STM100C, BIOPAC Systems Inc.) and current isolation unit (STMISOC, BIOPAC Systems Inc.) with a minimum of 10 seconds interval between stimuli. Recording EMG electrodes were positioned as described above and a ground electrode was placed on the distal radius over the styloid process, with all wires secured using thin open-cell foam wrap to reduce noise. Electrical stimulation was applied starting with a low voltage and gradually increased until the maximum H-reflex (H_max_) was visualized, and further increased until the maximum muscle response (M_max_) reached a plateau. Three measures of H_max_ were averaged and normalized to the average of three measures of M_max_ to calculate the H:M ratio, which can be interpreted as the proportion of the entire motoneuron pool capable of being recruited [37].

#### Corticospinal Excitability.

AMT (% maximal stimulus output) was recorded to quantify corticospinal excitability of the upper trapezius, middle deltoid, and flexor carpi radialis using a single-pulse TMS paradigm. Motor evoked potentials (MEPs) were elicited in each muscle using a magnetic stimulator (MagStim BiStim^2^, MagStim Company, Ltd., Wales, UK) with stimulation delivered via a 70 mm diameter figure-of-eight coil.

Participants were seated in an upright position in a height-adjustable chair with their hips flexed to 85° and a belt worn at the pelvis throughout the testing. Participants wore a Lycra swim cap with straight lines from the nasion to inion and bilateral from external auditory meatus to the vertex. A 1-cm x 1-cm grid was drawn on each swim cap to aid in locating the TMS coil properly over the primary motor cortex (M1) [38]. The coil was positioned over the contralateral cortical hemisphere in the area of M1 and was shifted by 1 cm in each direction over the grid to identify the optimal stimulating location (hotspot) [39]. The hotspot was defined as the site at which the greatest peak-to-peak MEP was evoked for the muscle tested at 50% maximal stimulator output.

Over the hotspot, the stimulus intensity was gradually reduced to determine the AMT. AMT was defined as the minimum stimulation intensity needed to evoked a measurable MEP (> 100 µV) during a tonic muscle contraction [40]. We determined the AMT using the TMS Motor Threshold Assessment Tool (MTAT 2.0) available online at: http://www.clinicalresearcher.org/software.htm. This program estimated AMT via a maximum-likelihood parameter estimation by sequential testing (PEST) procedure [41].

Participants were instructed to raise the tested arm to 90° in the scapular plane with the elbow extended and the forearm pronated (shoulder abduction) without external loading to collect MEPs for the upper trapezius and middle deltoid muscles, which elicited EMG amplitudes at approximately 15% MVIC EMG in the upper trapezius (observed activation: 14.6 ± 4.4%) and 20% MVIC EMG in the middle deltoid (observed activation: 20.0 ± 4.3%) to facilitate EMP acquisition [17]. Participants maintained isometric shoulder abduction as each stimulus was delivered, then relaxed and lowered their arm between stimuli. TMS recordings for the upper trapezius and middle deltoid were performed separately, although we utilized the same position and procedure. For the flexor carpi radialis, participants were seated with the shoulder flexed at 30°, the elbow flexed at 45°, and the tested forearm supported posteriorly on a padded armrest in the supinated position. Participants were instructed to maintain the wrist in a neutral position while loosely holding the dumbbell which produced 3–6% MVIC EMG in the flexor carpi radialis in healthy individuals as the stimuli were delivered, and then to relax following each stimulus [42]. A previous study yielded good reliability for AMT of the upper trapezius (ICC_3,1_: 0.81, MDC_95_: 3.89%), middle deltoid (ICC_3,1_: 0.89, MDC_95_: 2.60%), and flexor carpi radialis (ICC_3,1_: 0.87, MDC_95_: 2.57%) [43].

### Limb symmetry

Bilateral measurements were used to calculate the limb symmetry index (LSI) for each outcome as:


LSI = (Involved/Uninvolved) x 100\]

### Statistical analysis

In the current study, we estimated the sample size aiming to detect at least moderate linear bivariate correlation (Pearson’s *r* ≥ 0.60) with each patient-reported outcome, which required 15 (one-tailed) participants at α ≤ .05 and 1- β ≥ .80 (G*Power, version 3.1.9.6). Therefore, we estimated that 16 patients would be acceptable.

All data were assessed for normality (Shapiro-Wilk ≥ .05, skewness < |1.0|, kurtosis < 3) prior to analysis. Pearson’s product-moment correlation coefficients (*r*) were used to identify the relationships between objective measures of upper extremity neuromuscular function and patient-reported outcomes when data were normally distributed. Spearman rank correlation coefficients (ρ) were used in the event of non-normally distributed data. The relationships between neuromuscular function and patient-reported function were assessed separately for each PRO using the outcome measures from the involved limb and their respective LSIs. The absolute values of correlation coefficients were classified as negligible (0.0–0.30), low (0.30–0.50), moderate (0.50–0.70), high (0.70–0.90), or very high (0.90–1.0) [44]. Significantly correlated variables, including sample demographics (if more than one variable were present for a given PRO), were entered into a multiple linear regression model using a backward stepwise approach to determine the amount of variance the variables explained in each PRO. The adjusted *R*^*2*^ was also reported for each model. We assessed bivariate correlations between all significant predictor variables to rule out multicollinearity (*r* > 0.8). If multicollinearity was detected, the variable with the greater correlation coefficient was included.

All statistical analyses were performed using SPSS (version 25.0, IBM, Chicago, IL) and evaluated at an alpha level of 0.05.

## Results and discussion

Participants’ demographics are summarized in Table 1.

**Table 1:** Participant demographics and patient reported outcomes (mean ± standard deviation).

	Labral Repair Group (N = 16)
Sex	Male: 13, Female: 3
Age (years)	24.1 ± 5.0*
Height (cm)	179.1 ± 8.0
Mass (kg)	85.3 ± 19.1
Limb affected: Dominant/Non-dominant (%)	8/8 (50.0)
Time since surgery (months)	36.7 ± 33.3*
Tegner activity scale: before surgery	7.9 ± 1.0* (median = 8)
Tegner activity scale: current	6.3 ± 1.0* (median = 6)
Pain (Visual Analog Scale, cm)	0.27 ± 0.14
DASH (0: no symptoms – 100: worst symptoms)	5.3 ± 4.2
OSS (12: full function – 60: no function)	14.7 ± 2.3*
VR-12	
PCS (0: worst – 100: best)	55.1 ± 5.5
MCS (0: worst – 100: best)	48.8 ± 1.7*

Abbreviations: DASH, Disability of Arm, Shoulder and Hand; OSS, Oxford Shoulder Score; VR-12, Veterans Rand 12-Item Health Survey; PCS, Physical Component Score; MCS, Mental Component Score

*Non-normally distributed (Shapiro-Wilk Test ≤ 0.05)

**Fig 1:**
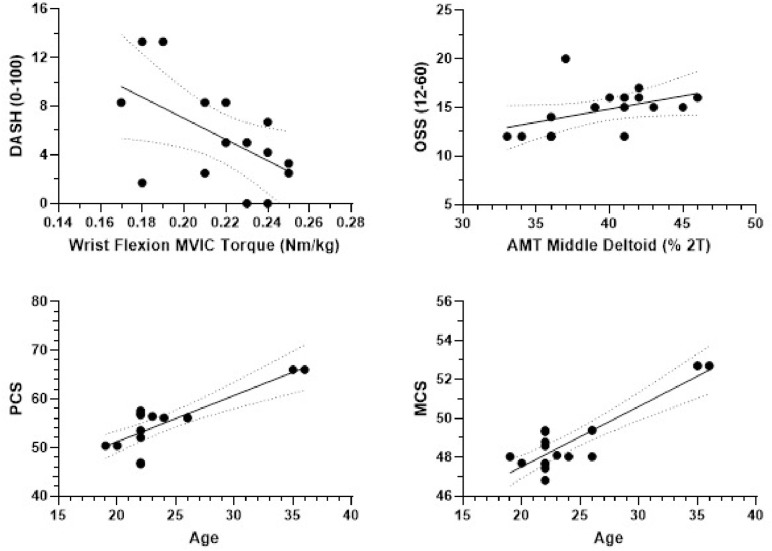
Relationship between patient-reported outcomes and muscle function or demographics.

Lesser wrist flexion MVIC torque moderately associated with higher DASH scores (*r* = −.523, *p* = .045). Higher AMT of the middle deltoid moderately associated with higher OSS (ρ = .570, *p* = .021). Higher age (ρ = .623, *p* = .010), lesser current physical activity level (ρ = −.821, *p* < .001) and lesser pain (*r* = −.499, *p* = .049) associated with higher PCS, while the strength of their associations varied. Higher age is also moderately associated with higher MCS (ρ = .550, *p* = .027). Limb symmetry indices did not associate with PROs (Table 2, Fig 1).

**Table 2:** Association between upper extremity muscle function, patient demographics, and patient-reported function.

	Correlation Coefficient (Spearman’s ρ)							
	DASH		OSS		VR-12 (PCS)		VR-12 (MCS)	
	Involved limb	LSI	Involved limb	LSI	Involved limb	LSI	Involved limb	LSI
Neuromuscular outcomes								
Shoulder abduction MVIC torque (Nm/kg)	−.278	−.089*	.052*	−.049*	.433	.156*	.225*	−.247*
Wrist flexion MVIC torque (Nm/kg)	**−.523**	−.019	−.239*	−.145*	.110	.371	−.239*	.127*
H:M ratio	−.104*	.032*	−.276*	.006*	−.373*	.054*	−.428*	−.460*
AMT upper trapezius (%−2.0T)	.152	.100*	.450*	.079*	.387	−.391	.126*	.412*
AMT middle deltoid (%−2.0T)	.214	.414	.570*	.443*	.160	−.096	−.118*	−.148*
AMT flexor carpi radialis (%−2.0T)	−.002	−.053	.320*	.129*	.032	−.118	−.235*	−.120*
Demographics								
Age (years)	−.176*		−.060*		.623*		.550*	
Tegner activity scale: current	.195*		−.040*		−.821*		−.366*	
Pain (VAS, cm)	.253		.117*		**−.499**		−.125*	
Time since surgery (months)	−.167*		−.131*		.433*		.272*	

Abbreviations: DASH, Disability of Arm, Shoulder and Hand; OSS, Oxford Shoulder Score; VR-12, Veterans Rand 12-Item Health Survey; PCS, Physical Component Score; MCS, Mental Component Score; LSI, Limb Symmetry Index; MVIC, maximal voluntary isometric contraction; AMT, active motor threshold; VAS, visual analog scale

*Spearman rank correlation coefficient (ρ)

**Bold:** Significant at *p* ≤ .05

Multiple regression was performed using age, current activity level, and pain given their independent associations with PCS. Although age and current activity level were significantly correlated, the magnitude of correlation was less than 0.8 (ρ = .790, *p* < .001). Thus, all those significantly correlated predictor variables were entered into a multiple linear regression model. All models that included those predictor variables were significant. However, the model including age and current activity level explained 73.2% of variance observed in PCS (adjusted *R*^*2*^ = .732, *p* < .001), which more effectively explained physical health than any other combination of predictor variables (Table 3). No combination of neuromuscular outcomes was input into a multiple regression model since only one variable associated with DASH and OSS, respectively.

**Table 3:** Multiple regression (backward stepwise) for PCS (VR-12) model estimation.

		Variance characteristics				Model characteristics		
	Predictor	Unstandardized β coefficient	Standardized β coefficient	ΔR^2^	*p* value	R^2^	Adjusted R^2^	*p* value
Model 1	Age	.668	.584	.692	.007	.780	.725	<.001
	Activity level	−1.713	−.311	.076	.120			
	Pain	−4.715	−.124	.020	.439			
Model 2	Age	.689	.602	.692	.004	.768	.732	<.001
	Activity level	−1.970	−.358	.076	.006			

Abbreviations: PCS, physical component score; VR-12, The Veteran’s Rand 12 Item Health Survey

### Discussion

We hypothesized that greater unilateral or symmetrical strength and desirable neural function in the affected limb would be associated with better patient-reported shoulder and upper extremity function and perceived global health. However, only wrist flexion strength and corticospinal excitability of the middle deltoid were associated with region-specific function among individuals nearly 3 years removed from glenohumeral labrum repair. Furthermore, no objective measures of upper extremity neuromuscular function were associated with global health. Younger individuals who were more physically active with greater pain demonstrated a worse perception of their global health, although the strength of association varied. Limb symmetry indices of any outcome did not associate with patient-reported function, which suggests that unilateral muscle function may drive patient perceptions of their physical function. Our results also show that factors associated with patient-reported outcomes vary in individuals with glenohumeral labral repair. This may suggest that a single factor does not comprehensively explain patient perceptions and emphasizes the needs for multifaceted clinical approaches to evaluate and treat individuals with glenohumeral labral repair.

Lesser wrist flexion strength in the affected limb associated with higher DASH scores, suggesting that strength deficits around the wrist may inform worse patient perceptions of upper extremity function after labral repair. We hypothesized that lesser shoulder abduction strength would inform perceptions of upper extremity and shoulder function to a greater extent than wrist flexion strength, yet this was not observed. Although limited evidence exists, a previous study [45] reported small-to-moderate correlations between muscle strength and patient-reported function, where lesser shoulder abduction, flexion, extension, internal rotation and external rotation strength each associated with worse region-specific function as measured by the American Shoulder and Elbow Surgeons Shoulder Outcome Survey in adults with rotator cuff tears. The lack of association between DASH score and region-specific muscle function observed in this study may be explained by the fact that participants only reported minimal pain in the involved shoulder during participation. Also, the DASH can potentially overestimate function allowing consideration of compensatory movement strategies using the contralateral limb or whole segments of the upper extremity to perform certain tasks, which may not be able to explain the conditions in the injured joint only. Although this is possible, we did not observe associations between strength and OSS, either.

We observed a mean DASH score of 5.3 ± 4.2 among individuals with glenohumeral labral repair, which may indicate our participants had minimally perceived disability at the time of enrollment. A 10-point difference in DASH score may be considered as a minimal clinically important difference [10,46], and a DASH score ranging from 0 to 29 was thought by most respondents to be the point where patients were not considering their upper-limb disorder a problem [47]. This result may potentially be explained by time from surgery and change in their activity levels. Our participants were an average of 3 years out of surgery, and at the time of enrollment, their activity levels were lower than preinjury by an average of 1.6 points. While we did not ask participants questions regarding previous and current physical activities, reductions in physical activity engagement or altering the type of activities from demanding overhead tasks to those less demanding may contribute to better (lower) DASH scores among them. Although no outcomes associated with time from surgery, limited evidence has shown improvements in DASH can occur over the first two years from surgery [10]. suggesting that an earlier time from surgery might demonstrate different relationships. Collectively, considering that the DASH questionnaire assesses self-rated overall upper extremity disability and symptoms [9] rather than perceived function of affected shoulder itself [26], this result may simply suggest that compensatory function in muscle groups distal to the affected shoulder helps adequate level of activities in daily living (e.g., fastening buttons, brush teeth) listed in the questionnaire in this population.

We observed a moderate association between lesser corticospinal excitability of the middle deltoid and worse perceived function of the operated shoulder as measured by the OSS, suggesting that lesser cortical drive to a prime mover of the shoulder may influence the way individuals perceive their shoulder function. Lesser corticospinal excitability during low-level voluntary muscle contraction of the deltoid has been documented in patients with chronic rotator cuff tears in literature [17], which aligns with our results. Corticospinal pathways play an important role in the initiation of movement and sustained motor control [48,49], which include delicate control of force, precision of movement, angulation, rate of change of movement and muscle tension [48]. Our results may indicate that impaired neural excitability is associated with ineffective force production or less coordinated movements, which in turn is related to worse perceived function. Notably, the lack of association between maximal shoulder abduction strength and perceived function of the operated shoulder, despite its relationship with impaired neural function, may indicate that precise voluntary motor control has greater influence on perceived function rather than muscle strength itself. Low OSS (14.7 ± 2.3) scores, suggestive of satisfactory joint function among those underwent arthroscopic surgery for rotator cuff, subacromial impingement or degenerative inflammatory conditions [26], were observed in our participants. Although the OSS is sensitive to clinical change [29], evidence regarding a minimal clinically important difference is lacking [26], which limits our interpretation of the observed scores. Overall, lesser corticospinal excitability was associated with worse shoulder function among individuals nearly 3 years removed from glenohumeral labrum repair, suggesting that centrally mediated neuromuscular function may be meaningful for improved perception of regional health.

Administering global health measures allows clinicians to measure domain specific health changes (e.g., physical, mental, social) beyond the direct target of the intervention. PCS reflects individual’s perceived physical function, energy, pain, and role limitation due to physical health [50]. Low-to-high correlations were observed between perceived physical health and demographic variables (age, current activity level, and pain). However, we did not observe significant association between measures of muscle function and physically related global health, which challenges our hypothesis. Greater pain at the end of strength testing from session 1 associated with worse PCS, suggesting pain response is still a typical problem even among individuals nearly 3 years removed from glenohumeral labrum repair.

Higher activity level was associated with worse PCS, suggesting patients may experience lower quality of life when they are more active. This finding may appear counterintuitive at face value, where more physically active individuals are expected to have better perceived physical health. However, the results may indicate a need to consider how individuals perceive their physical health differently based on activity level between healthy and injured populations. For example, patients may be fearful of returning to demanding physical activities when recovering from glenohumeral injury or surgery, such that remaining less active or modifying their activities contributes to better perception of health. Previous studies have reported lower current health-related quality of life among former high-level collegiate athletes compared to non-collegiate athletes [51,52], suggesting that highly structured, competitive athletic participation may result in injuries that persist into adulthood and possibly contributing to reductions in activity. This was also seen in our study with lower current activity levels compared to their pre-injury, thereby lowering their health-related quality of life. Thus, future research that investigates the association between physical activity level and perceived physical health and well-being in injured populations may need to take fear avoidance behaviors into consideration.

A previous systematic review [53] documented that 83% of patients who underwent type II SLAP repair were satisfied with their physical function and activity level 2–4 years after surgery. However, satisfaction rates are reportedly lower in competitive athletes including overhead athletes, where only 68% report an excellent level of satisfaction at an average of 2–3 years after surgery [7]. Our participants were highly active before surgery (median Tegner = 8) and included 7 current or former intercollegiate athletes (43.8% of sample), which may suggest they are more aware of their physical limitations resulting from previous injuries, thus having a substantial influence on long-term health [52]. In contrast, if people who are less active routinely perform minimally demanding activities of daily living, this may help explain why their perception of their physical health is high because they’re able to easily accomplish their normal tasks. Although speculative, this explanation may have contributed to our observation of higher activity levels inversely associated with level of satisfaction in physically related global health. In other words, it may be reasonable to consider differences in activity demand for the level of satisfaction after a glenohumeral labral repair in future studies.

Pain, age, and current activity level were all associated with physically related global health independently, however, multiple regression identified the model including age and current activity level explained 73.2% of variance observed. Among those variables, older age was the strongest predictor of better physical health score. This finding may appear to contradict normal expectations of age-related trends in health perceptions. In physically active individuals, physical health scores generally decrease with increasing age [54–56]. However, age-related declines in perceived physical health were not reported until 45 years or later among the general population in previous studies compared to our much younger participants (24.1 ± 5.0 years, range: 18−36) with glenohumeral labral repair. As mentioned, our data suggest that lower activity levels are associated with better perceived physical health but older age (ρ = −.745, *p* = .001). Although speculative, better perceptions of physical health may be explained by lower activity level to some extent in individuals with glenohumeral labral repair, while true relationship between age and perceived physical health remain unclear particularly in relatively younger injured population. Overall, our results suggest that only emphasizing muscle function may not improve physically related global health. Considering that age was the strongest predictor for perceived physical health but not a modifiable factor, pain management is still important from a patient perspective.

Higher age was associated with better perceived mental health in our study, indicating that younger patients reported lower mentally related global health. The MCS reflects individuals’ feelings or emotions, and how emotional problems interfere with personal productivity [50], yet it remains unclear how patients’ age influences their perception of global health. Although examining associations between age and activity level is beyond the scope of our study, the results might imply more active younger individuals reported lower levels of satisfaction regarding their global health following surgery due to insufficient mental maturity or lack of coping skills. No measure of muscle function was associated with perceived mental health, which may suggest global health, especially mentally related global health, is more driven by psychosocial or contextual factors, and thus it is difficult to improve global health only by focusing on muscle function.

### Clinical implication

Patient-reported outcomes for regional and global health play important roles in contemporary health care practice. The results of this study suggest several factors that are important during clinical care of those with a history of glenohumeral labral repair.

Unilateral wrist flexion strength and centrally mediated neuromuscular function of the middle deltoid may influence perceived upper extremity and shoulder function for individuals approximately 3 years from glenohumeral labral repair. The observed association between greater wrist flexion strength and better DASH score suggests the importance of intervening on regions adjacent and distal to the injured joint to target improvements in the function of the entire upper extremity after glenohumeral labral surgery. On the other hand, centrally mediated deltoid activation appears to be more related to perceptions in shoulder specific function, which may provide clinicians with a novel route to target the neural origins of muscular impairments from a patient’s perspective. However, no measure of symmetry in upper extremity neuromuscular function was associated with perceived region-specific function in this population. Future studies may investigate PROs in response to interventions that enhance neuromuscular function at supraspinal level or target muscular strength in regions adjacent to the injured joint.

Even though age is not a modifiable factor, it was associated with perceived global health. Our results may highlight a need for clinicians to consider younger patients as vulnerable population who are more susceptible to exhibiting poor psychological responses to injury, and to adjust clinical approaches for this population as needed. Especially for those who participate in competitive sport, the long-term risks of diminished health-related quality of life need to become a priority for patients and clinicians. For example, interventions such as physical activity transition programs need to be explored to help them transition from highly structured and competitive athletics to lifestyle physical activity [52]. In order for age-specific approaches to be available, further research investigating factors closely associated with individuals’ perception of global health for targeted age groups is warranted. Also, pain management appears to remain an important consideration to optimize individuals’ perception of their global health.

Overall, our results can be an important first step to develop evidence-based assessment and intervention strategies for individuals with glenohumeral repair, where clinicians should address the factors that are closely associated with perceived function and global health.

### Limitation

The cross-sectional design of our study limits inference on causality between neuromuscular function and patient-reported outcomes. Our sample was different based on time from surgery (mean: 36.7 months, range: 6–120 months), sex (male: 13, female: 3), and types of surgery (SLAP: 9, Bankart: 7). Time after surgery was not associated with any neuromuscular or patient-reported outcomes in our study. Prior work [10,57,58] has also reported weak-to-moderate, but statistically non-significant associations between time from arthroscopic shoulder surgery and change in DASH scores from 6 to 21 months post-surgery; the natural history of DASH and OSS scores beyond 21 months from surgery has yet to be explored. With a wide distribution of time from surgery, lack of associations appears to suggest those outcomes do not improve linearly over time, which may highlight the need for early intervention. However, the potential long-term impact of surgery on neuromuscular function, patient reported outcomes, and the association between them remain unclear from our study. To address this knowledge gap, future longitudinal studies would aid clinicians to further understand temporal changes in neurophysiology and patient reported outcomes following glenohumeral labral repair. Also, there is no previous clinical evidence regarding differences in subjective and objective function by sex or types of surgery in this population, necessitating future research.

We may have been underpowered to detect relationships of all magnitudes. While bivariate correlations were powered to detect relationships of 0.6 or stronger with our sample, we detected relationships as low as 0.499. Also, the small sample size (n = 16) might limit the generalizability of the results from multiple regression analyses despite the adjusted coefficient of determination (adjusted *R*^*2*^) accounting for model overfit to present the individual contributions of age and activity level to perceived physical function and well-being. While our data identified that age explained more than 70% variance, one-third of the variance remained unexplained. This suggests that our data are better suited for generating hypotheses rather than confirming them, and that a larger sample size would be needed to clarify any unique combination of factors underlying the presence or absence of an association with perceived physical function.

Lastly, we must interpret PRO scores with caution. Our participants appeared to be a high-functioning sample based on the DASH score and OSS. It is possible that lower-functioning individuals would demonstrate different relationships. Therefore, in future studies, differences in physical activity demand need to be taken into consideration when evaluating subjective function in individuals with a history of glenohumeral labral repair.

## Conclusion

Involved limb wrist flexor strength, corticospinal excitability of the middle deltoid, age, pain, and activity level explained patient-reported upper extremity function and global health for the individuals with a history of glenohumeral labral repair. These results support the inclusion of muscle strength both in regions adjacent and distal to the injured joint, as well as upper extremity neural function in assessment and intervention practices to improve patient-reported functional outcomes. Despite the limited sample size, older age, less pain, and lower activity level associated with better physical health scores, which suggests the importance of developing age-appropriate and physical activity-specific clinical interventions for individuals with glenohumeral labral repair. While age was the strongest predictor of physical health scores, considering it is not a modifiable factor, it would still be practically reasonable to formulate interventions guided by pain management to improve physically oriented quality of life. Taken together, clinicians can use the information from this study to suggest factors that may be meaningful to patients who seek care and guidance following glenohumeral labral repair.

## Supporting information

S1 Fig Relationship between patient-reported outcomes and muscle function or demographics. Plots describe the relationship between wrist flexion strength of the affected limb and self-reported upper extremity disability and symptoms (top left), corticospinal excitability of the affected deltoid and perceived change of functional status in the operated shoulder (top right), age and perception of physical health (bottom left), and age and perception of mental health (bottom right).Abbreviations: DASH, Disability of Arm, Shoulder and Hand; MVIC, maximal voluntary isometric contraction; OSS, Oxford Shoulder Scores; AMT, active motor threshold; PCS, physical component scores; MCS, mental component scores.
(TIF)

S1 Table Participant demographics and patient reported outcomes (mean ± standard deviation). (DOCX)

S2 Table Association between upper extremity muscle function, patient demographics, and patient-reported function. (DOCX)

S3 Table Multiple regression (backward stepwise) for PCS (VR-12) model estimation. (DOCX)

S1 File Demographics and outcome measures. The spreadsheet presents demographic and outcome measures from the participants with history of glenohumeral labral repair (N = 16) that were used for analyses in the current study. Labels: mvc_MD_inv, mass normalized maximal shoulder abduction torque in the involved limb; mvc_MD_cont, mass normalized maximal shoulder abduction torque in the contralateral limb; mvc_FCR_inv, mass normalized maximal wrist flexion torque in the involved limb; mvc_FCR_cont, mass normalized maximal wrist flexion torque in the contralateral limb; H:M_inv, H:M ratio of flexor carpi radialis in the involved limb; H:M_cont, H:M ratio of flexor carpi radialis in the contralateral limb; amt_UT_inv, active motor threshold of upper trapezius in the involved limb; amt_UT_cont, active motor threshold of upper trapezius in the contralateral limb; amt_MD_inv, active motor threshold of middle deltoid in the involved limb; amt_MD_cont, active motor threshold of middle deltoid in the contralateral limb; amt_FCR_inv, active motor threshold of flexor carpi radialis in the involved limb; amt_FCR_cont, active motor threshold of flexor carpi radialis in the contralateral limb; DASH, Disability of Arm, Shoulder and Hand; OSS, The Oxford Shoulder Score; PCS, physical component score; MCS, mental component score; LSI_mvc_MD, limb symmetry index of maximal shoulder abduction strength; LSI_mvc_FCR, limb symmetry index of maximal wrist flexion strength; LSI_H:M, limb symmetry index of H:M ratio; LSI_amt_UT, limb symmetry index of active motor threshold in the upper trapezius; LSI_amt_MD, limb symmetry index of active motor threshold in the middle deltoid; LSI_amt_FCR, limb symmetry index of active motor threshold in the flexor carpi radialis.
(XLSX)
