# Supplementary figures and images for: Relationships between upper extremity neuromuscular function and patient-reported outcomes among individuals with a history of glenohumeral labral repair
Source: PLoS One. 2025 Dec 12;20(12):e0338260. doi: 10.1371/journal.pone.0338260 (PMC12700448; doi:10.1371/journal.pone.0338260)

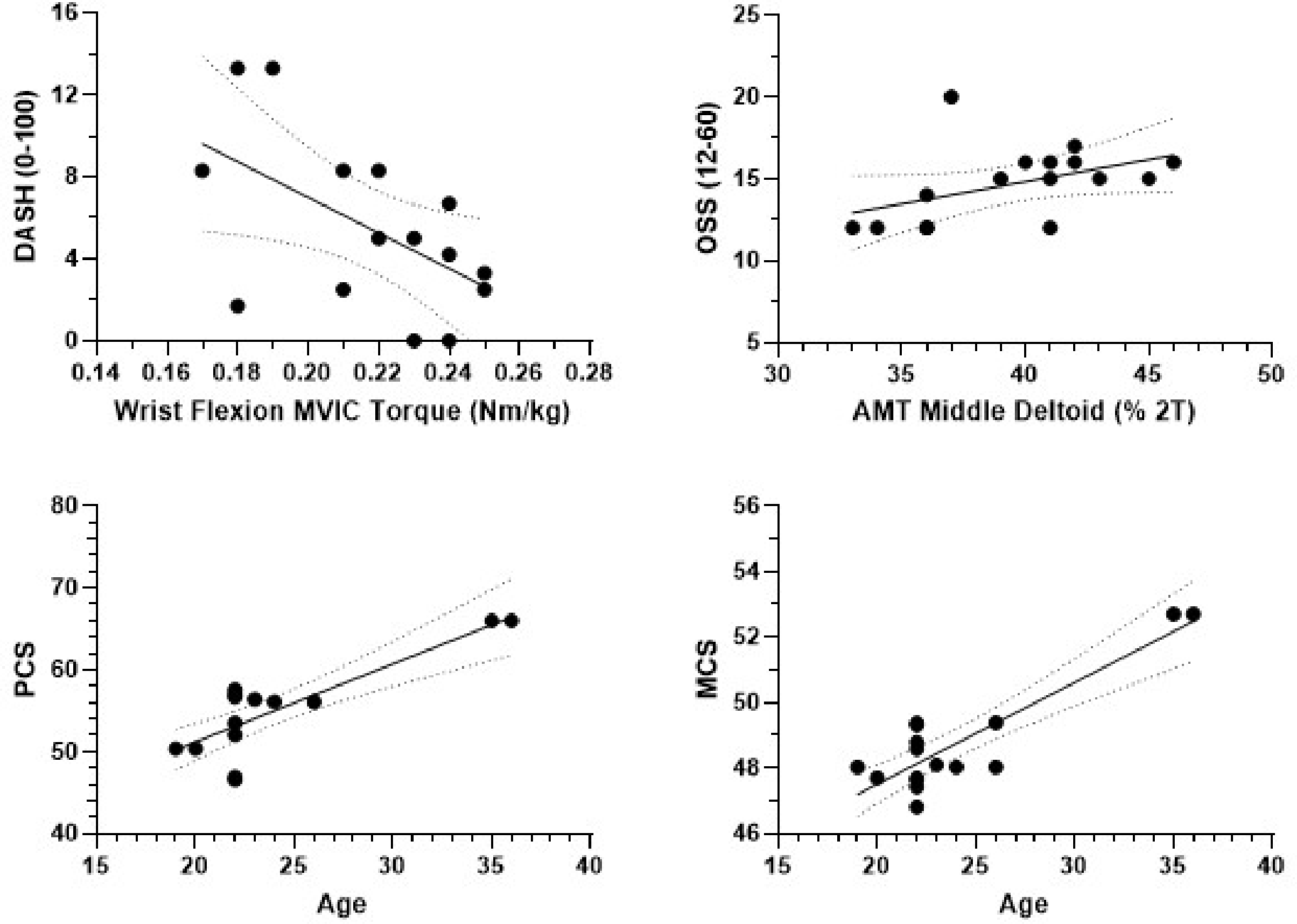

Supplement: S1 Fig — Plots describe the relationship between wrist flexion strength of the affected limb and self-reported upper extremity disability and symptoms (top left), corticospinal excitability of the affected deltoid and perceived change of functional status in the operated shoulder (top right), age and perception of physical health (bottom left), and age and perception of mental health (bottom right).Abbreviations: DASH, Disability of Arm, Shoulder and Hand; MVIC, maximal voluntary isometric contraction; OSS, Oxford Shoulder Scores; AMT, active motor threshold; PCS, physical component scores; MCS, mental component scores. (TIF) [file pone.0338260.s001.tif]
