# Supplementary material for: Relationships between upper extremity neuromuscular function and patient-reported outcomes among individuals with a history of glenohumeral labral repair
Source: PLoS One. 2025 Dec 12;20(12):e0338260. doi: 10.1371/journal.pone.0338260 (PMC12700448; doi:10.1371/journal.pone.0338260)
Supplement: S1 Table — (DOCX) [file pone.0338260.s002.docx]

| Table 1: Participant demographics and patient reported outcomes (mean ± standard deviation) | | |
| --- | --- | --- |
|  | Labral Repair Group (N= 16) | |
| Sex | Male: 13, Female: 3 |  |
| Age (years) | 24.1 ± 5.0* |  |
| Height (cm) | 179.1 ± 8.0 |  |
| Mass (kg) | 85.3 ± 19.1 |  |
| Limb affected: Dominant/Non-dominant (%) | 8/8 (50.0) |  |
| Time since surgery (months) | 36.7 ± 33.3* |  |
| Tegner activity scale: before surgery | 7.9 ± 1.0* (median = 8) |  |
| Tegner activity scale: current | 6.3 ± 1.0* (median = 6) |  |
| Pain (Visual Analog Scale, cm) | 0.27 ± 0.14 |  |
| DASH (0: no symptoms - 100: worst symptoms) | 5.3 ± 4.2 |  |
| OSS (12: full function - 60: no function) | 14.7 ± 2.3* |  |
| VR-12 |  |  |
| PCS (0: worst - 100: best) | 55.1 ± 5.5 |  |
| MCS (0: worst - 100: best) | 48.8 ± 1.7* |  |
| Abbreviations: DASH, Disability of Arm, Shoulder and Hand; OSS, Oxford Shoulder Score; VR-12, Veterans Rand 12-Item Health Survey; PCS, Physical Component Score; MCS, Mental Component Score | | |
| *Non-normally distributed (Shapiro-Wilk Test ≤ 0.05) | | |
