# Supplementary material for: Relationships between upper extremity neuromuscular function and patient-reported outcomes among individuals with a history of glenohumeral labral repair
Source: PLoS One. 2025 Dec 12;20(12):e0338260. doi: 10.1371/journal.pone.0338260 (PMC12700448; doi:10.1371/journal.pone.0338260)
Supplement: S2 Table — (DOCX) [file pone.0338260.s003.docx]

| Table 2: Association between upper extremity muscle function, patient demographics, and patient-reported function | | | | | | | | |
| --- | --- | --- | --- | --- | --- | --- | --- | --- |
|  | Correlation Coefficient (Spearman’s ρ) | | | | | | | |
|  | DASH | | OSS | | VR-12 (PCS) | | VR-12 (MCS) | |
|  | Involved limb | LSI | Involved limb | LSI | Involved limb | LSI | Involved limb | LSI |
| Neuromuscular outcomes |  |  |  |  |  |  |  |  |
| Shoulder abduction MVIC torque (Nm/kg) | -.278 | -.089* | .052* | -.049* | .433 | .156* | .225* | -.247* |
| Wrist flexion MVIC torque (Nm/kg) | **-.523** | -.019 | -.239* | -.145* | .110 | .371 | -.239* | .127* |
| H:M ratio | -.104* | .032* | -.276* | .006* | -.373* | .054* | -.428* | -.460* |
| AMT upper trapezius (%-2.0T) | .152 | .100* | .450* | .079* | .387 | -.391 | .126* | .412* |
| AMT middle deltoid (%-2.0T) | .214 | .414 | **.570*** | .443* | .160 | -.096 | -.118* | -.148* |
| AMT flexor carpi radialis (%-2.0T) | -.002 | -.053 | .320* | .129* | .032 | -.118 | -.235* | -.120* |
| Demographics |  |  |  |  |  |  |  |  |
| Age (years) | -.176*  .195*  .253  -.167* | | -.060*  -.040*  .117*  -.131* | | **.623***  **-.821***  **-.499**  .433* | | **.550***  -.366*  -.125*  .272* | |
| Tegner activity scale: current |  |  |  |  |  |  |  |  |
| Pain (VAS, cm) |  |  |  |  |  |  |  |  |
| Time since surgery (months) |  |  |  |  |  |  |  |  |
| Abbreviations: DASH, Disability of Arm, Shoulder and Hand; OSS, Oxford Shoulder Score; VR-12, Veterans Rand 12-Item Health Survey; PCS, Physical Component Score; MCS, Mental Component Score; LSI, Limb Symmetry Index; MVIC, maximal voluntary isometric contraction; AMT, active motor threshold; VAS, visual analog scale | | | | | | | | |
| *Spearman rank correlation coefficient (ρ) | | | | | | | | |
| **Bold:** Significant at *p* ≤ .05 | | | | | | | | |
