# Supplementary material for: Relationships between upper extremity neuromuscular function and patient-reported outcomes among individuals with a history of glenohumeral labral repair
Source: PLoS One. 2025 Dec 12;20(12):e0338260. doi: 10.1371/journal.pone.0338260 (PMC12700448; doi:10.1371/journal.pone.0338260)
Supplement: S3 Table — (DOCX) [file pone.0338260.s004.docx]

| Table 3: Multiple regression (backward stepwise) for PCS (VR-12) model estimation | | | | | | | | | |
| --- | --- | --- | --- | --- | --- | --- | --- | --- | --- |
|  |  | Variance characteristics | | | |  | Model characteristics | | |
|  | Predictor | Unstandardized β coefficient | Standardized β coefficient | ΔR^2^ | *p* value |  | R^2^ | Adjusted R^2^ | *p* value |
| Model 1 | Age | .668 | .584 | .692 | .007 |  | .780 | .725 | < .001 |
|  | Activity level | -1.713 | -.311 | .076 | .120 |  |  |  |  |
|  | Pain | -4.715 | -.124 | .020 | .439 |  |  |  |  |
|  |  |  |  |  |  |  |  |  |  |
| Model 2 | Age | .689 | .602 | .692 | .004 |  | .768 | .732 | < .001 |
|  | Activity level | -1.970 | -.358 | .076 | .006 |  |  |  |  |
| Abbreviations: PCS, physical component score; VR-12, The Veteran’s Rand 12 Item Health Survey | | | | | | | | | |
